# Notes on the Treatment of Bronchitis

**Published:** 1896-06-13

**Authors:** Solomon C. Smith

**Affiliations:** Physician to the Westminster General Dispensary


### Medical Progress and Hospital Clinics.

[*The Editor will be glad to receive offers of co-operation and contributions from members of the profession. All letters should be addressed to* The Editor, at the Office, 428, Strand, London, W.C.]

### NOTES ON THE TREATMENT OF BRONCHITIS.

By Solomon C. Smith, M.D., M.R.C.P., Physician to the Westminster General Dispensary.

II.

Bearing in mind then the conditions in which bronchitis is likely to occur (as a result of chill to the body, of direct irritation of the mucous lining of the bronchial tubes, or as a complication of other diseases), the disturbances of function which it is likely to set up (altered secretion, difficulty of expectoration, interference with respiration), and the secondary pathological consequences which are likely to follow it (thinning of tubes, bronchiectasis, collapse of lung, lobular pneumonia, emphysema, dilatation and hypertrophy of the heart, and general sapræmia from absorption of septic products from the tubes, and ultimately from the infected lungs), the question is what can we do to modify the course of the disease and prevent its evil effects?

By drugs directly modifying the inflammatory process it is to be feared that we can do but little, for even such drugs as are imagined to have a direct influence upon the bronchial mucous membrane hold a far less important part in the treatment of the disease than some imagine. Truly by alkalies, iodide of potassium, antimony, ipecacuanha, squills, and certain terebinthine and balsamic medicines, we can modify the secretion of the tubes and their underlying glands; and by various stimulants of the respiratory centre and of the circulation, of which strychnine, ether, ammonia, and alcohol may be taken as examples, and antispasmodics, such as opium, belladonna, lobelia, and stramonium, we can overcome or lessen certain functional difficulties which arise in the course of the disease; but this is a very different thing from directly influencing the inflammatory process, in doing which it will be found that expectorants and medicines specially esteemed for their action in pulmonary complaints have far less efficacy than many more general measures, although they are useful enough in dealing with its after results.

As then in inflammation of so many other tissues what we have to do in bronchitis is (*a*) to remove the cause of the disease, (*b*) to lessen the strain put upon the bronchial tubes in the performance of their function while the disease lasts, and (*c*) to assist in removing the products of the disease and in preventing its evil consequences. It is usually the case that by the time the patient comes under treatment the apparent cause, or at least one cause, has already been removed. The chill to the surface, or the damp fog which has been the obvious exciting cause of the attack has no doubt passed away, but few things come from single causes, and while it is true that an exposure to cold or the breathing of a smoky fog will often determine the onset of the disease, it is also certain that some constitutional state is often the reason of its effectiveness in so doing. Gout, kidney disease, heart disease, rickets, and a crowd of acute maladies, prominent among which are whooping-cough, measles, and typhoid fever, are common causes of bronchitis, as also we should add are certain insanitary conditions, which as well as acting as predisposing causes, play a very definite part in determining the type of, and the course which will be taken by, the disease, and there are certain overloaded conditions of the digestive organs which, while normal enough in many who are actively engaged in work, are efficacious causes of the continuance of an inflammation which has once been set up.

In acute cases it almost always happens that the patient, taken from a life of activity, has an intestinal canal charged with an amount of material quite inappropriate for the invalid life which he will have to lead until his bronchial tubes recover their normal state. It is well, then, in such cases to commence treatment by a purge, not on account of any effect it may be supposed to have upon inflammation, but as a means of placing the patient in the best position for recovery.

In chronic cases also it will often be found that the patient, from his shortness of breath, his dread of cold, and his morbid fear of producing cough, which he looks on as his worst enemy, has stayed in the house and taken but little exercise, and that until the resulting general torpor of his chylo-poietic viscera has been overcome, it is impossible to produce any impression upon his bronchial disorder.

In both these circumstances mercury is of the greatest service; in acute cases a single fairly large dose of calomel, in chronic ones repeated doses of blue pill combined with either rhubarb or colocynth.

Gout complicates bronchitis in various ways. Bronchitis may actually be a form of acute gout, or it may be an extension downwards of an acute gouty pharyngitis. In either of these cases the treatment is that of acute gout; potash and colchicum, or salicylate of soda and ammonia, according to the idiosyncrasies of the patient. But the less pronounced forms of gout may cause the persistence of a frequently recurring form of chronic bronchitis; while, again, a subacute form of bronchitis may alternate with a gouty eczema or urticaria, and in both these cases it may be necessary to have recourse to careful dietetic treatment or to the use of saline waters, or to hydrotherapeutics in their wider sense, including the use of Turkish or Russian baths. But wherever gout is found to be at the root of the disease the treatment should be carried on during the intervals between the attacks as well as during their continuance; exercise in the open air and careful attention to the eliminative organs being the principal points to be attended to. In fact, it may well be believed that in many cases in which change of climate works such marvels as it undoubtedly does sometimes in elderly bronchitis, it does so by means of the outdoor exercise which it renders possible, in far larger degree than by virtue of any influence the better air may have upon the bronchial mucous membrane.

